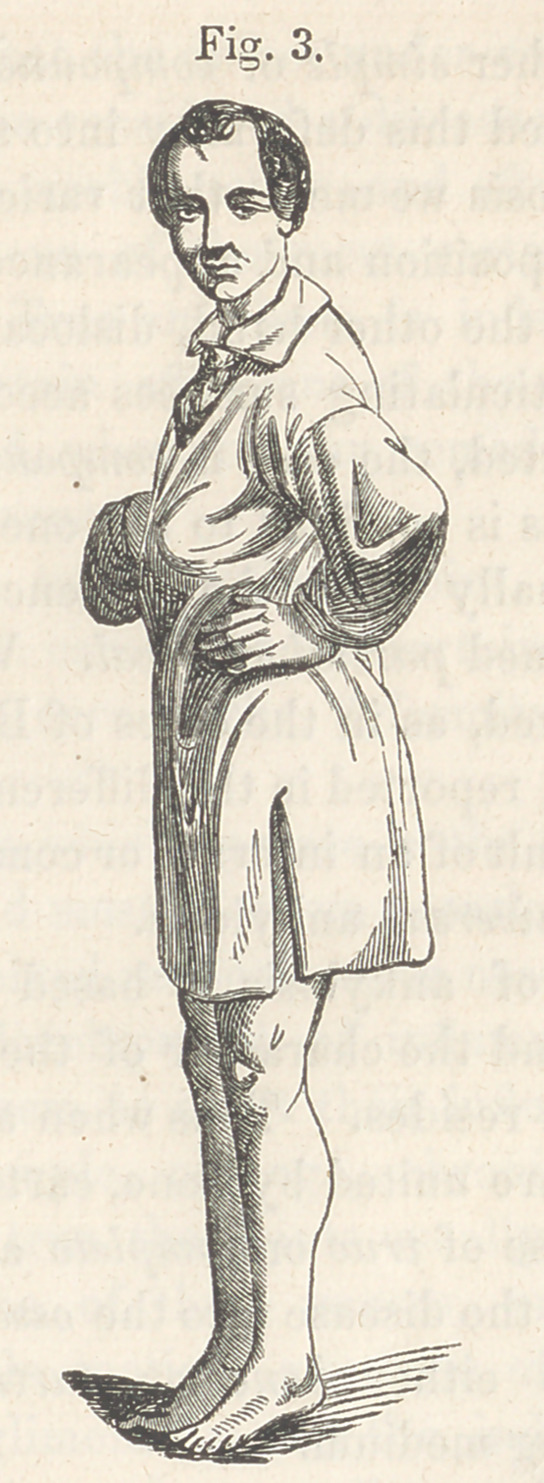# Clinic of the Jefferson Medical College

**Published:** 1851-01

**Authors:** C. W. Hornor

**Affiliations:** Senior Clerk of the Surgical Department


### *Clinic of the Jefferson Medical College. Service of* Profs. Mütter and Pancoast.

Report furnished by C. W. Hornor, M. D., Senior Clerk of the Surgical Department.

*Cases treated from September 1st to December 21st.*

| | |
|---|---|
| Whole number of cases, - - - | 906 |
| " " operations, - - | 120 |

Viz., Lithotomy, 1; Amputation, 7, (viz., leg, 1, fingers, 4, Toes, 2;) necrosis, 4, (viz., femur, 1, arm, 1, foot, 2;) trephining Cranium for epilepsy resulting from depressed fracture, 1; Castration, 3; resection of femur for ankylosis, 2; screw applied for false ankylosis, 2, in one of which the hamstring tendons were divided; introduction of seton for pseudarthrosis of humerus, 1; extensive plastic operations for malignant ulcer of face, 3; do. for mercurial ulceration of cheek, 2; do. for burn of arm, 1; removal of fungoid tumour of Scalp (very large,) 1; reduction of luxated humerus, 2, one of seven weeks standing; for stricture of œsophagus, 2; trephining for serocystic tumor of lower jaw, 1; contraction of tendon of pectineus muscle treated by subcutaneous section, 1; extirpation of the eye, 1; cataract, 2; tumours, 19, (viz., arm, 2, brow, 1, cheek, 2, face, 1; cancer of lips, 2, back, 1, sarcomatous of epigastrium, 1, condylomatous, 2, epithelial of tongue, 1, cancroid of ear, 1, epithelial of face, 1, fatty in groin, (very large,) 1, epulis, 1, erectile of lip, 1, steatomatous on nose, 1, fibrous on the neck, 1;) removal of foreign bodies, (glass from hand, 1, slug from thigh, 1, shot from hand 3,) distichiasis, 1; strabismus, 4; abscess, 8, (viz., over sternum 2; head, 1; groin, 1; face, 1; cheek, 1; psoas, 1;) paronychia, 4; pterygium, 3; excision of tonsils, 3; ganglion on wrist, 3; removal of cartilage in knee joint, 1; staphyloma corneæ, 2; fistula lachrymalis, 4; do. in ano, 3; do. in urethra, 1; hydrocele, 3; hare lip single and double, 2; scirrhous mamma, 1; stricture of urethra, 1; evacuation of humors of eye, 1; cicatrix of hand treated by subcutaneous section of tendons, 1; exostosis of tibia, 1; fissure of tongue, 1; injection of eustachian tube, 1; introduction of seton in scrotum for hydrocele, 1; aneurism by anastomosis, 2, one embracing one half of head and extending into the roof of the mouth; varicocele 3; polypus of nose, 3; onychia, 1.

In addition, the apparatus for club foot, spinal disease, and trusses for hernia, were applied in several cases.

Days of operating 32; average number of patients each day, 28.

### *Clinic of October 23d*, 1850.

*Cases presented.*—Varicose veins of leg; Caries of sternum; Ulcer of breast; Hemorrhoids; Spina bifida; Coxalgia; Scrofulosis; Tertiary syphilis; Ulcer of leg; Fistula lachrymalis; Neuralgia of eye; Cancer of cheek; Rupture of biceps fascia; Conjunctivitis simplex; Granular conjunctivitis; Paronychia; Oblique hernia; Acute osteitis of carpal bones; Abscess of neck; Contusion of spine; Tumor of thigh; Chronic conjunctivitis; Ankylosis of knee; Necrosis of femur; Caries of spine; Elephantiasis of leg; Psorophthalmia; Rheumatic inflammation of the eye; Epilepsy from depressed fracture; Cancer of lip—operation; True ankylosis of knee.* Total, 32.

* The names of the patients were given, but have been omitted for want of room.—Eds.

Several of these cases were exhibited before the class and prescribed for or operated upon; after which Prof. Mütter read the case of John Mahony, performed the operation of "resection," proposed by Dr. J. R. Barton, and concluded with some remarks on "ankylosis."

Case 32. *True ankylosis of the knee, following acute inflammation of the joint.*—The patient, John Mahony, æt. 31, a robust sailor, was taken on the 6th day of January, 1849, without any apparent cause, with an acute pain in the right knee. The inflammation which followed soon disabled him, and he was obliged in consequence to apply at the Boston Marine Hospital for surgical assistance. Notwithstanding judicious treatment, suppuration in and about the joint supervened, and to evacuate the pus the joint was twice opened. After remaining in the hospital seven months and a half, during the whole of which period the knee continued to discharge copiously, he left the institution with the leg flexed at nearly a right angle, and with no motion in the affected joint. It appears that he next entered the alms house, and from this institution, after a month or two, he passed into the hospital on Quarantine Island. Thus it would seem that several months elapsed between the development of the attack and its termination in complete ankylosis. When he presented himself at the Jefferson College clinic in April, 1850, no trace of inflammatory action remained. The integuments exhibited here and there a cicatrix, but were otherwise healthy, while not the slightest motion could be effected in the knee-joint. The general health and spirits of the patient were excellent, and he earnestly besought us to attempt some operation for the relief of his deformity, a deformity utterly incapacitating him for duty in his vocation. Fearing the effect of long confinement, in warm weather, he was advised to return again in the fall, when something would be attempted for him. Accordingly he made his appearance in October, 1850, and on the 23d inst., in the presence of several medical gentlemen and the class, the operation was performed by Prof. Mütter, aided by Prof. Pancoast and Drs. Goddard and Wallace. The appearance of the limb is well shown in the annexed cut. The measure selected was that proposed by

Dr. J. R. Barton, and the various steps of the operation may be grouped together under five heads.

Fig. 1.

1. *Incision of the integuments.*—In this, the direction of Dr. Barton was carried out. Two incisions were made over the

Fig. 2.

femur, just above the patella. The first commenced at a point opposite the upper and anterior margin of the *inner* condyle of the femur and passing obliquely across the front of the thigh, terminated on the outer side just below the centre of the limb. The second incision commenced also on the inner side, about two inches and a half above the first; and passing likewise obliquely across the thigh, terminated with the other in an acute angle. (See fig. 2.) By these incisions were divided the integuments, the tendon of the extensor muscle of the leg, at its insertion into the upper part of the patella, and some of the contiguous fibres of the rectus crureus muscle themselves, a greater part of the rectus internus, and a portion of the rectus externus muscles. A flap composed of these tissues was thus raised from the femur close to the condyles. The soft parts were next detached from the bone, so as to admit of the use of a saw. Attention was then directed to the hemorrhage, (comparatively slight,) and, this arrested, the second step of the operation was executed.

2. *Sawing the Bone.*—This is the most important step in the whole operation, for if we commit an error in calculation, a successful result cannot be anticipated. Dr. Barton has well explained that the *wedge shape* is the only one proper to be given to the piece of bone removed, and again, that the *angle* of section must be neither too *acute*, nor too *obtuse.* If *too acute*, the cut surfaces would reach each other before the limb is sufficiently extended; if *too obtuse*, the leg would bear full extension without entirely closing the gap, and hence reunion might be prevented. To determine the *angle* then is a matter of great consequence and to do this the plan proposed by Dr. Goddard is the best, viz.: *Take the angle of deformity, and then remove from the bone the complement of this angle.* Having determined this point, previously, the flap was held out of the way, and a triangular piece of bone, of the proper size, (see fig. 2,) removed by means of a small narrow-bladed saw. The incisions did not extend through the bone, but united within a few lines of its posterior portion, leaving this to be separated in another manner.

3. *Breaking the bone.*—The solution of continuity was rendered complete by simply bending the leg backward, the thin portion of bone breaking without difficulty. This, the third step in the operation, is important, and was introduced by Dr. Barton to protect the popliteal artery from the danger of being wounded by the saw, and also to steady the fragments of the divided bone by the interlocking of the fractured surfaces. Without this interlocking, the artery would be much endangered by the *sharp ends* of the divided bone coming in contact with it during the subsequent attempts to straighten the limb.

4. *The dressing.*—The flap was next adjusted, maintained in place by suture and strap, and the cold water dressing applied, the limb being carefully held at its original angle of deformity.

5. *Application of splint.*—Dr. Barton used a double-inclined plane, upon which the limb was placed, during the subsequent treatment, but Dr. Mütter employed a screw of Stromeyer's, modified slightly, for the reason that the extension of the limb could be made with more rapidity, and also because it allows the ham to be entirely free from pressure (a most important point) during the whole treatment. In this splint the limb was placed at the *angle of deformity*, no extension being made for fear of forcing the rough ends of the bone into the ham, the patient put to bed lying on his back, and a strict antiphlogistic treatment ordered, with anodynes in case of pain.

On the tenth day, the screw was turned and extension of the leg commenced. Extension was postponed to this period in order that the asperities of the fragments might be removed, or at least embedded in plasma, and thus occasion no inconvenience or hazard in the future treatment. Each day the extension was increased, by simply turning the screw a few threads, and at the expiration of six weeks the leg was found perfectly straight, the cut surfaces united (see fig. 2) and shortened about a quarter of an inch, just sufficient to give the heel the proper elevation for convenient use. During the entire period of treatment not a bad symptom was manifested, the patient having experienced little or no pain, and the wound uniting by *first intention*, with the exception of the acute angle of the flap, which ulcerated.

Fourteen weeks have elapsed since the day of operation, and the union is firm and perfect, the limb presenting the appearance shown in the annexed cut. (Fig. 3.)

Fig. 3.

*Remarks.*—The term *ankylosis** is derived from the Greek word, signifying *bent* or *crooked*, and is employed to designate that condition of a joint in which its motions, both active and passive, are either partially or entirely destroyed, accompanying which loss there is usually a change in the natural shape of the part. It must be borne in mind, however, that often in ankylosis there is little or no alteration of the shape of the joint involved; it is neither *bent* nor *crooked*, as the etymology of the term would indicate. The beautiful specimens of ankylosed hip, elbow, and knee joints, contained in my collection, and to which your attention has already been directed on another occasion, prove this fact. As the stiffness may involve *one* joint or *several*; may depend on different conditions of the constituents of the part,

* Ankylosis, or anchylosis, ἡ αγχυλωσὶς, (ἀγχύλη, a bending, flexure, more properly the bend of the elbow, the ham, a contracted joint, "contractos articulos ἀγχυλας, Græci nominant." Cels. v. 28.) Stiffness, rigidity, and adhesion of a joint, distortion.

and may also be either *simple* or *compound, partial* or *complete*; surgeons have divided this deformity into several kinds.

By *simple* ankylosis we mean that variety in which the joint involved assumes a position and appearance as nearly natural as possible ; when, on the other hand, dislocation, either partial or complete, of the articulating surfaces accompanies the stiffness, or the limb is distorted, the case is *compound* or *complicated.*

When the stiffness is confined to but one articulation, and is dependent, as it usually is, on the influence of some local cause, the ankylosis is termed *partial* or *local.* When all, or nearly all the joints are involved, as in the cases of Baron Percy, Bernard Connor, and others, reported in the different surgical works, and the defect is the result of an internal or constitutional cause, it is called *general* or *universal* ankylosis.

Another division of ankylosis is based upon the degree of motion preserved, and the character of the tissue in which the resistance to motion resides. Thus when all motion is lost, and the articular facets are united by bone, cartilage, or dense fibrous tissue, we have a case of *true* or *complete* ankylosis. Mayo has divided this form of the disease into the *osseous, cartilaginous* and *mixed,* inasmuch as either *bone* or *cartilage,* or *both* together may form the uniting medium.

When stiffness is dependent upon some defect of the soft parts of the joint, either *extra capsular* or *intra-capsular,* or of the *capsule* itself, and the joint is susceptible of slight motion at the time our examination is made, or if this be absent, capable of being rendered moveable by proper subsequent management, the ankylosis is *false, or incomplete.* Again, these false ankyloses may be divided into the *extra-capsular, intra-capsular,* and *capsular,* and this division, so far as prognosis is concerned, is one of much importance. In the first, the tissues surrounding the joints, viz., integuments, cellular tissue, muscles, tendons, and fascia, are involved, and the case is generally susceptible of cure. In the second and third, the rigidity is occasioned by some lesion of the ligaments or synovial membrane, and, although curable if taken in time, is excessively prone to terminate in incurable, or true ankylosis, by involving the cartilages, or bones, or both, in the disease.

It must be obvious that the defect under consideration cannot, with strict propriety, be considered a disease; it is rather a product or termination of morbid action, and often, on this account, its occurrence proves one of the most certain indications of a cessation of disease. True ankylosis is indeed often the only favorable result in certain affections of the joints, and we consider the case as cured, when, by our remedies, we are able to bring about this occurrence.

Before undertaking to relieve stiff joint, therefore, we must carefully investigate its causes, and ascertain whether or not it is proper to attempt a cure of the deformity to which it often gives rise.

*Causes.*—The causes which operate in the production of ankylosis are numerous, and most of them occasion the complaint by keeping the parts involved motionless, or nearly so, for a length of time, or by the development of inflammation. There are some, however, that seem to exert their influence under all circumstances; for example, old age, chronic rheumatism, and chronic gout. It is true the joints usually involved from the operation of any one of these causes, are those possessing comparatively but little motion, as those of the spine, pelvis, and some of the ginglimoidal; and the individuals themselves are forced to lead very sedentary lives, which will of course favor the occurrence of stiffness.

True ankylosis is also occasionally developed in the effort which nature makes to protect herself from harm in certain curvatures of the spinal column. This beautiful provision is well shown in several specimens in my collection, taken from individuals affected with the complaint. Ledges of bone are thrown into the hollow of the different curves, so that the spinal column is supported, and prevented from yielding so far as to endanger life.

True ankylosis is, however, most generally the result of some disease of the synovial membranes, cartilages, or bones of a joint, although it may result from long confinement to one position, as has been clearly shown by Malgaigne, Tessier, and others. It is commonly believed that the ankylosis resulting from long confinement of a joint to one position, as in the treatment of fractures of the extremities, belongs to the class of incomplete or false ; and this, to a certain extent, is true, but we often have produced instead, complete and incurable stiffness. This fact adds another to the long list of objections to the use of immoveable apparatus, in the treatment of fractures.

False ankylosis, especially the extra-capsular form, is developed by causes somewhat similar, although they produce lesions by no means so grave as those which are present in the true. The most common of all these causes is rest. When a joint, even although perfectly healthy, is confined to one position for any length of time, we find its integuments, and the cellular tissue beneath them, contract, and become more or less rigid; its ligaments and tendons also stiffen, and the muscles to which they are attached shorten where the limb is flexed, and lengthen where it is extended, and often undergo changes in their tissue, by which they lose their physical characteristics, and are rendered, to a certain extent, useless, while the synovial secretion is arrested or diminished. A good illustration of the influence of rest upon the joints, is afforded by the stiffened limbs of the Fakirs of India, who, actuated by religious motives, condemn themselves to the observance of one position during their whole lives. The same thing is often seen after the treatment of fractures, luxations, or sprains, when the limb has been maintained in one position for too long a period.

Wounds followed by sloughing of the skin, burns, &c. ; in short, any injury likely to be followed by extensive cicatrization, may lay the foundation of extra-capsular ankylosis. You all know the power with which one of these cicatrices forms, often destroying or displacing some of the most important parts of the body. Here is a hand, the fingers of which nearly touch the back of the forearm, in consequence of a burn. This drawing you recollect as the one taken from a patient whose chin was drawn down to the sternum, by the cicatrix of a burn, and held there for twenty-seven years. Such cases can only be relieved by very extensive plastic operations.

Extra-capsular ankylosis is also sometimes brought on by the development of an ulcer, or an abscess, or a phlebitis, or an angioleucitis, the result of some trivial wound or blow received upon the part. The complaint being generally confined to one side of the limb, there is contraction in this direction, produced partly by the patient's flexing the limb to take off pressure from the inflamed surface, and partly by the swelling itself, displacing the tendons and fascia in the neighborhood. By and by lymph is deposited, and unless the disease is cured, there remains permanent contraction of the limb, with motion in but one direction. This form is readily distinguished from that dependent upon shortening of the muscles, by the following experiment. If we take hold of the limb farthest removed from the body, and steady the one to which it is attached, and then attempt to separate them, or bring the member to its proper shape, we find in this form of ankylosis that the soft parts, although rigid and unyielding, are yet smooth and even upon the surface. Now, when the tendons or muscles are chiefly in fault, and the same experiment is tried, we find them standing out in bold relief, as hard and as rigid as pieces of wire, while the soft parts in the vicinity are comparatively loose and yielding.

Different affections of the tendons or muscles, in the vicinity of a joint, may also cause extra-capsular ankylosis. A man, for example, receives a slight wound of a tendon or muscle, irritation or even inflammation of the part sets in, and the joint contracts by a shortening of the muscle alone. Sometimes the same cause may excite adhesions between the tendon and its sheath, or may even cause a sloughing of the same part.

Sometimes this form of ankylosis is dependent on a constitutional cause, as rheumatism or gout. Often, too, a loss of muscular power, either by sloughing, or wounds, or paralysis on one side of the joint, by destroying, as it does, the balance of power which naturally exists, will bring on this form of ankylosis.

The sound muscles being no longer opposed by their antagonists, pull the limb into an unnatural position; and as there is no power to overcome this influence, they keep it contracted, and gradually accommodate themselves in length to the altered condition of the parts, so that when we attempt to straighten the member we find it impossible to do so, until by stretching or division with the knife, the shortened muscles are made to yield.

In certain forms of club foot, torticollis, contraction of the fingers and toes, &c., this kind of ankylosis is encountered. In cases of long standing, we invariably find the muscles and tendons of the weak side stretched and frayed out, and so much weakened that months and even years may elapse before they regain their natural tone and vigor.

This should always be explained to the patient, who will be obliged to make use of artificial support for some time after the member has been restored to its natural shape.

Extra-capsular ankylosis is also the result of contraction of fasciæ, as we see in certain forms of contracted elbow, knee, and hands. It may also proceed from the growth of tumors, and the deposit of bone around the joint.

*Capsular ankylosis.*—This form is generally the result of a severe strain or twist of the joint, in consequence of which the capsular ligament is more or less injured; a luxation or fracture near the joint, gout, rheumatism, wounds, and even rest may occasion the same thing. It is extremely difficult to distinguish this variety of ankylosis from the *intra-capsular;* but usually in the latter, the cause operating is more violent, the stiffness is accompanied with more pain, and it is more difficult to move the joint. When a capsular ankylosis is examined by dissection, we find the fibrous tissue of the capsule thickened and hardened, and sometimes converted here and there into cartilage or bone.

*Intra-capsular ankylosis* is the result of some disease of the synovial membrane, usually acute or chronic inflammation, or their results; but the peculiar degeneration of this tissue, so well described by Sir Benjamin Brodie, may also lay the foundation of the complaint. The bond of union here, is nothing more than organized coagulable lymph, which sometimes stretches across the joint from bone to bone, in bands, or cords; at others it is deposited in patches, and, in a few rare instances, has been found spread over nearly the whole of the articulating surfaces of the joint, thus glueing the bones, as it were to each other.

The history of the case will generally enable us to decide as to the precise nature of the bond of union; but it should always be borne in mind that synovitis may lay the foundation of capsular, or even extra-capsular ankylosis, producing, as it often does, subinflammation of the cellular and fibrous tissues about the joint, and also the peculiar contraction of the muscles and tendons usually met with in diseases of the articulation.

It also frequently gives rise to true ankylosis—the disease extending to the cartilage and bones. The extreme rigidity of the joint, the apparent soundness of the tissues around it, the swelling which in the first stage of the disease, is usually present, the pain excited by moving the articulation, or pushing one bone against another, the character of the cause, and the fact that such cases are usually preceded by all the symptoms of inflammation of the synovial membranes, all serve to indicate to us the variety of the affection.

*Morbid Anatomy.*—From the explanation already given of the different varieties of ankylosis, the appearances on dissection may be readily imagined. But there sometimes exist certain effects to which reference has not yet been made. When resulting from inflammation we find all the products of this lesion manifested in one or more of the tissues. We may have, for example, simple *thickening* of the soft tissues, with *muscular contraction and adhesion* from effusion of plasma both within and without the articulation; deposits of calcareous matter in these bands of plasma, constituting the *intermediate ankylosis* (*l'anchylose par intermède,*) of some authors; destruction of cartilage, and *fusion* of the bones (" *l'anchylose par fusion,*") and again when the osseous deposite is abundant we have often a kind of socket formed on the outside of the joint into which one of the bones is received, and which has been described by Cruveilhier as *l'anchylose par invagination.* With these changes in the constituents of the joint, we have often subluxation and distortion and wasting of muscles. When *rest* is the cause of ankylosis, we have less alteration of the bony tissue of the joint, although here as under other circumstances, attrition of cartilages and fusion of bones may take place. The soft tissues, however, are always atrophied, and the muscles have been known in cases of long standing to undergo the *adipose transformation.* This is a rare result, but I have seen it in several instances.

*Liability.*—It is usually stated that the ginglimoid articulations are more liable than the orbicular to both true and false ankylosis; and the observation is correct, owing chiefly to the circumstance that the former are more exposed to accidents.

Their large articulating surfaces, and the number of tendons and fascia by which they are surrounded, also predispose to the occurrence of the disease.

*Diagnosis.*—There is scarcely any difficulty in distinguishing the different varieties of ankylosis from other complaints, but it often demands a great deal of tact to distinguish them from each other. False ankylosis, for example, may exist, and yet the joint be as immovable as in the true variety; but by careful examination we may generally arrive at a just diagnosis. If, for instance, there is no motion in the joint, when it is twisted or turned with great force, if these efforts excite no pain, if the stiffness has been preceded by extensive intra-capsular disease, if the joint is comparatively but little swollen, if the patient feels a jarring when the limb is struck, or when he takes a false step, if it be in the leg, if he dreads these shocks, and finally, if, when we attempt either to bend or straighten the limb, the muscles and tendons about it are scarcely moved, we may pretty safely conclude that we have a case of true ankylosis to contend with. False ankylosis is also sometimes confounded with a rigid condition of the muscles surrounding a joint, which suffers from some acute or chronic inflammatory disease. A case of this kind was some years since shown me by my friend Dr. Rodman, and it occurred in the son of Mr. B——— of Schuylkill Eighth street. This lad had suffered from coxalgia for some months, and his limb was shortened seven inches, the thigh flexed at a right angle with the pelvis, and no perceptible motion in the hip joint. At first I was under the impression that true ankylosis had taken place, but on a more careful examination it struck me that it might probably be a case of simple false ankylosis from rigid muscles. I therefore applied an apparatus by which moderate and constant extension of the muscles might be kept up, and had the satisfaction to find that in the course of a few days, without pain, fever, or any inconvenience, the limb was reduced to the plane of its fellow, and within one inch of its natural length. The details of this case will be given on another occasion. The fact that this condition of the muscles may give rise to complete immobility, should be constantly borne in mind in an examination of stiffened joints.

In forming a diagnosis between the different kinds of false ankylosis, we must take into consideration the history of the case. If the rigidity is the result of rest, cicatrices, affections of the sub-cutaneous cellular tissue, spasm of the muscles, slight wounds of the tendons, or injury of the fascia, it is probably extra-capsular. If caused by a spasm, or punctured wound, or a blow, it is capsular, and, finally, if acute or chronic synovitis, or gout, or rheumatism, or slight disease of the cartilages or bones has existed, we shall have the intra-capsular form.

*Prognosis.*—The prognosis in this affection varies with the character of the lesion, whether it be true or false; and if false, the nature of its cause, the duration of the case, the age and health of the patient, and the joint involved.

The prognosis as regards cure is almost always unfavorable in true ankylosis; and, until very recently,—indeed until the publication on this subject by Dr. J. Rhea Barton, of Philadelphia,—all such cases were ranked among the incurable forms of the affection, and even now the propriety of resorting to the operation of Dr. Barton, must be the result of mature deliberation. Many cases give rise to so little inconvenience as scarcely to warrant the hazard of the remedy; while others, as those that result from caries, or extensive disease of a joint, should not, as a general rule, be touched, inasmuch as ankylosis is the most favorable termination of the complaint; besides which, the operation may excite anew an inflammation in the part sufficiently intense to destroy the patient.

When the stiffness is partial the prognosis is usually more favorable, but even here we sometimes find it impossible to effect a cure. When it is dependent upon extra-capsular lesion, of no very long standing, and there is no loss of tendon or muscle by sloughing, the prognosis is favorable; but if the ankylosis be capsular or intra-capsular, unless the primary disease has been slight, or confined to the ligamentous tissue, as in gout or rheumatism, and the case is of recent occurrence, we shall generally have great difficulty in accomplishing our object.

When the stiffness depends on the location of a tumor, in the vicinity of a joint, the prognosis is generally favorable, inasmuch as we can remove the cause. The younger the person, the greater the probability of a cure in almost all cases, for, as we increase in years, the tissues become more rigid and unyielding. The general health, too, of the patient must be taken into consideration; there are some persons so irritable and prone to inflammation, that the slightest effort towards a cure, made either with the knife or mechanical means alone, is sure to excite disease. We should, therefore, carefully examine the case, and determine, as nearly as possible, the propriety of instituting any treatment before our attempts are commenced. In illustration of the importance of this care, I may mention that several persons have either lost their lives, or been reduced to a very critical position, by the attempts to cure ankylosis by the screw. In my own practice I have been obliged, in two cases, to suspend the treatment, until the general health was so much improved as to justify the attempt to straighten the limb to be renewed. The prognosis is also modified by the joint involved, for we find it much more easy, as a general rule, to cure ankylosis of the orbicular than of the ginglimoidal articulations. The function of the joint is also found to modify the prospects of cure: thus, when the articulations of the maxillary bones are involved, as in the cases of Cruveilhier and others, it will be found impossible, by any operation, to afford relief. In one case, mentioned by Blackburn, the patient died from inanition in consequence of this cause.

(To be concluded in our next.)

## Figures and Tables

**Fig. 1. f1:**
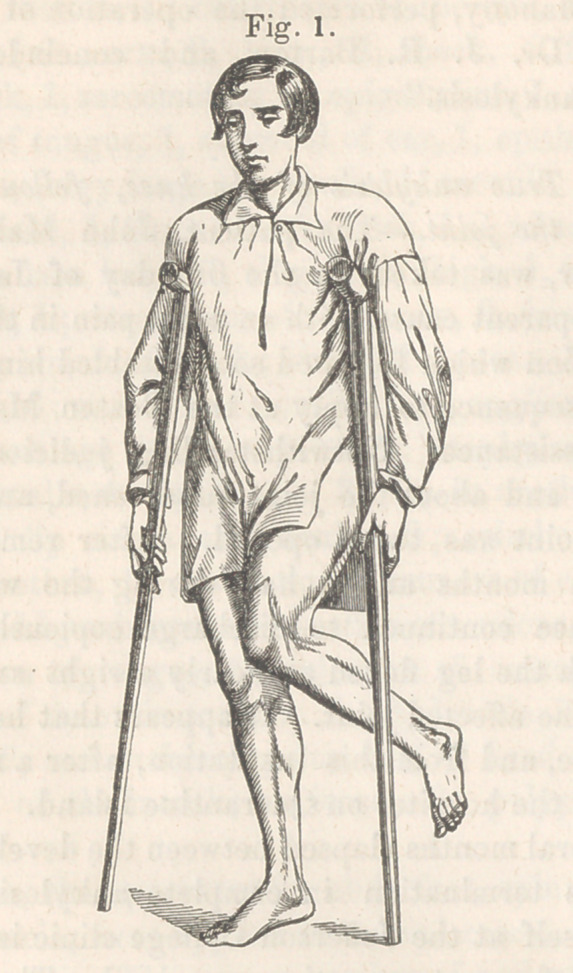


**Fig. 2. f2:**
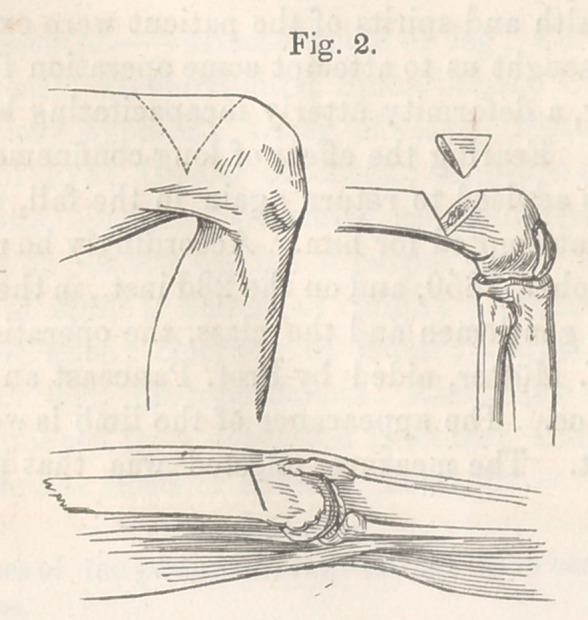


**Fig. 3. f3:**